# Neurofilament Light Chain (NfL) in Blood—A Biomarker Predicting Unfavourable Outcome in the Acute Phase and Improvement in the Late Phase after Stroke

**DOI:** 10.3390/cells10061537

**Published:** 2021-06-18

**Authors:** Milos Pekny, Ulrika Wilhelmsson, Anna Stokowska, Turgut Tatlisumak, Katarina Jood, Marcela Pekna

**Affiliations:** 1 Laboratory of Astrocyte Biology and CNS Regeneration, Center for Brain Repair, Department of Clinical Neuroscience, Institute of Neuroscience and Physiology, Sahlgrenska Academy at the University of Gothenburg, 40530 Gothenburg, Sweden; ulrika.wilhelmsson@neuro.gu.se; 2 Florey Institute of Neuroscience and Mental Health, Parkville, Melbourne 3010, Australia; 3 School of Medicine and Public Health, University of Newcastle, Newcastle 2308, Australia; 4 Laboratory of Regenerative Neuroimmunology, Center for Brain Repair, Department of Clinical Neuroscience, Institute of Neuroscience and Physiology, Sahlgrenska Academy at the University of Gothenburg, 40530 Gothenburg, Sweden; anna.stokowska@neuro.gu.se (A.S.); marcela.pekna@neuro.gu.se (M.P.); 5 The Stroke Research Center, Department of Clinical Neuroscience, Institute of Neuroscience and Physiology, Sahlgrenska Academy at the University of Gothenburg, 40530 Gothenburg, Sweden; turgut.tatlisumak@neuro.gu.se (T.T.); katarina.jood@neuro.gu.se (K.J.); 6 Department of Neurology, Sahlgrenska University Hospital, 41345 Gothenburg, Sweden

**Keywords:** neurofilament light chain, intermediate filament proteins, nanofilament proteins, biomarkers, stroke, cerebrovascular disease, acute phase after stroke, late phase after stroke, stroke recovery, stroke rehabilitation, synaptic plasticity, secondary neurodegeneration

## Abstract

Increased sensitivity of methods assessing the levels of neurofilament light chain (NfL), a neuron-specific intermediate filament protein, in human plasma or serum, has in recent years led to a number of studies addressing the utility of monitoring NfL in the blood of stroke patients. In this review, we discuss that elevated blood NfL levels after stroke may reflect several different neurobiological processes. In the acute and post-acute phase after stroke, high blood levels of NfL are associated with poor clinical outcome, and later on, the blood levels of NfL positively correlate with secondary neurodegeneration as assessed by MRI. Interestingly, increased blood levels of NfL in individuals who survived stroke for more than 10 months were shown to predict functional improvement in the late phase after stroke. Whereas in the acute phase after stroke the injured axons are assumed to be the main source of blood NfL, synaptic turnover and secondary neurodegeneration could be major contributors to blood NfL levels in the late phase after stroke. Elevated blood NfL levels after stroke should therefore be interpreted with caution. More studies addressing the clinical utility of blood NfL assessment in stroke patients are needed before the inclusion of NfL in the clinical workout as a useful biomarker in both the acute and the chronic phase after stroke.

## 1. Introduction

Stroke is the second most common cause of death globally [1,2]. One in six individuals will get a stroke during their lifetime, and in 10% of these, stroke will be the cause of death [1,2]. Most stroke victims live with long-term disabilities. Yet, the therapeutic options for stroke remain limited. Ischemic stroke is typically caused by an occlusion of an artery in the brain, either by a locally formed thrombus or by an embolus formed at a more distant place, such as the heart, and leads to the development of an ischemic infarct. Haemorrhagic stroke is caused by the rupture of a cerebral artery and subsequent bleeding. While intravenous thrombolysis or mechanical thrombectomy represent effective acute treatments for ischemic stroke, these therapies have to be administered within a narrow time window after stroke and only after the exclusion of cerebral haemorrhage, and thus are available only to a subset of ischemic stroke patients. Stroke-induced loss of function results from tissue loss and neuronal dysfunction within the tissue surrounding the stroke lesion as well as in other regions of the brain. Post-stroke functional recovery results from reversal of tissue dysfunction, functional remapping, cell genesis, angiogenesis and vascular remodelling, stroke-induced changes in neuronal connectivity, adaptive glial responses, and neural plasticity that leads to new connections via axonal sprouting and formation of new neuronal synapses [3,4,5,6,7].

Stroke survivors often suffer from long-term or permanent impairment in cognition and motor functions. Functional recovery that occurs in many stroke survivors is typically most pronounced during the first half year after stroke and can be facilitated by rehabilitative interventions [8,9]. Further improvement is possible in the late stage after stroke, in particular in conjunction with targeted rehabilitation [8,10,11]. A very important consequence of stroke is secondary neurodegeneration, which is triggered in cortical and subcortical regions distant but connected to the infarct area. Post-stroke secondary neurodegeneration continues for months or even years, contributing to cognitive decline and hampering long-term functional recovery [12,13,14,15].

Neurofilament light chain (NfL) is an intermediate filament protein that constitutes intermediate filaments, a part of the cytoskeleton, of neurons (intermediate filaments are sometimes referred to as nanofilaments). Together with the other four neuronal intermediate filament proteins, namely neurofilament heavy chain, neurofilament medium chain, alpha-internexin and peripherin, NfL assembles into neurofilaments, which are important for dendritic branching and growth and stability of axons in both central and peripheral nerves and for post-traumatic axonal regeneration [16,17,18]. Axonal damage leads to NfL release into the extracellular space, and hence, NfL levels in the cerebrospinal fluid or in peripheral blood serve as a biomarker of axonal damage and neurodegeneration in a range of neurological disorders including multiple sclerosis [19,20], Alzheimer’s disease, frontotemporal dementia, Parkinson’s disease, Huntington’s disease and amyotrophic lateral sclerosis [21,22].

## 2. High Blood NfL Levels in the Acute Phase after Stroke Predict Unfavourable Outcome

Already in the first days after ischemic stroke, blood NfL levels are increased and peak during the initial three months [23,24,25]. The actual levels of NfL in blood vary depending on the study population, study design, sampling protocol, sample handling and the assays used and are therefore not directly comparable between studies. The reported [23,24,25,26,27,28] median levels of NfL in blood samples obtained between 24 h and 3 months post-stroke range from 39 pg/mL [26] to 210 pg/mL of serum [24] as compared to 19 pg/mL and 32 pg/mL, respectively, in age-matched controls in those studies. In the acute phase after ischemic stroke, the blood levels of NfL were demonstrated to positively correlate both with the volume of the infarct [24,27] and with stroke-induced neurological deficit [26,27]. This link between infarct volume and acute-phase blood NfL levels is conceivably, at least partly, the explanation for why the aetiology of ischemic stroke seems to be an important determinant of blood NfL levels. Patients in the subgroups that in general present with larger stroke, i.e., those with stroke due to a large-vessel disease and cardioembolism, show the highest NfL levels in blood, both acutely and at 3-month follow-up [25]. The blood levels of NfL determined in the acute phase after stroke have also been shown to be of prognostic value: in the first 3 months after ischemic stroke, high blood NfL levels correlate with unfavourable clinical outcome, both short-term and long-term [24,25,26]. Blood NfL levels were increased in patients with newly evolved lesions at 3-month follow-up compared to the remaining patients with small subcortical infarcts (lacunar stroke due to cerebral small-vessel disease) both in the acute phase and after 3 months, pointing to blood NfL as a biomarker of active cerebral small-vessel disease [23]. A recent meta-analysis based on data from five clinical studies concluded that, compared to patients with lower blood NfL levels, patients with higher levels of NfL in blood samples obtained within the first 7 days after the ischemic stroke event had a 1.71-time higher risk of poor functional outcome during follow-up 3–6 months later [29]. Also, performance in activities of daily living was shown to negatively correlate with blood NfL levels at 3–5 months after stroke [27]. Thus, in the acute stage after stroke, blood NfL levels reflect the extent of ischemic injury and are predictive of unfavourable outcome, in particular during the first months.

## 3. Blood NfL as a Biomarker of Post-Stroke Secondary Neurodegeneration

Increased levels of NfL in cerebrospinal fluid and blood are found routinely in association with clinical progression of primary neurodegenerative diseases including Alzheimer’s disease, amyotrophic lateral sclerosis, Huntington’s disease and Charcot–Marie–Tooth disease (reviewed in [30]). Even in healthy individuals, the blood levels of NfL increase approximately 2.2% per year between the ages of 18 and 70 years [19,30]). Given that blood NfL levels show positive correlation with age both in healthy individuals [19] and in stroke survivors [24,31], it is conceivable that blood NfL levels after stroke are indicative of age-related neurodegenerative changes as well as of stroke-induced secondary neurodegeneration. Indeed, 6 months after stroke, the blood levels of NfL were shown to positively correlate with secondary neurodegeneration of major white matter tracts within the infarcted hemisphere as determined by diffusion tensor MRI [24]. As the association between blood NfL levels and secondary neurodegeneration remained after adjustment for age, sex, hypertension and recurrent ischemic lesions [24], these results point to the potential utility of blood NfL levels as a biomarker of stroke-induced secondary neuroaxonal injury that is independent of age.

## 4. Blood NfL as a Predictor of Functional Improvement in the Late Phase after Stroke

**Figure 1:**
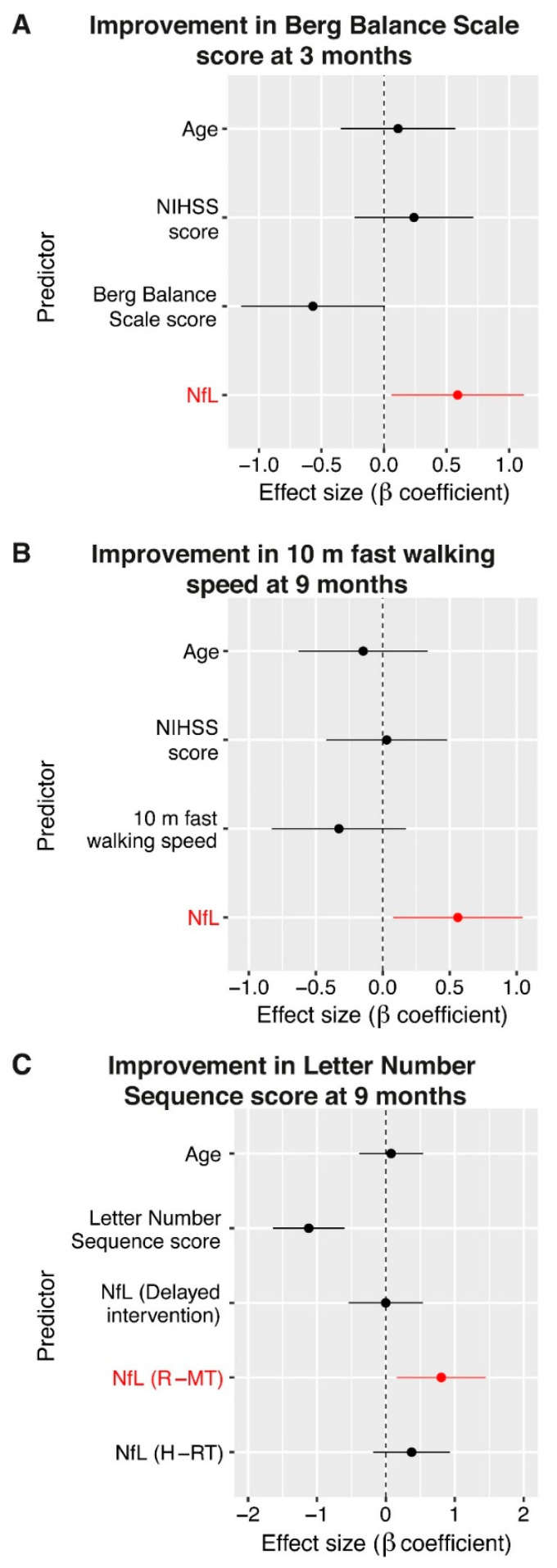
Blood neurofilament light chain (NfL) levels predict functional improvement in the late phase after stroke. In the cognitive domain, but not for balance and gait, the predictive value of elevated blood NfL levels was dependent on the type of intervention. (**A**) Forest plots showing estimated standardized coefficients and their 95% confidence interval for the predictors in fully adjusted logistic regression analysis, modelling functional improvement in balance, (**B**) gait and (**C**) cognition as a measure of the relative importance of each variable. NIHSS, NIH Stroke Scale. R-MT, rhythm- and music-based therapy; H-RT, horse-riding therapy. Study participants in the delayed intervention group did not receive any rehabilitation during the clinical trial but were offered rhythm- and music-based therapy after study completion. Reproduced from [31] under CC BY-NC 4.0.

There are only a few studies that measured blood NfL levels later than 6 months after stroke. Pedersen and co-workers found that when adjusted for age and cardiovascular risk factors, blood NfL levels of stroke survivors 7 years after stroke were not different from those in control subjects [25]. Similarly, blood NfL levels in individuals 15 months after stroke due to small-vessel disease were comparable to the levels in controls [23]. A longitudinal 9-year follow-up study of 503 subjects with small-vessel disease found baseline blood NfL levels to be associated with disease progression and both baseline and future small-vessel disease-related cognitive impairment, but not with the risk of dementia [28]. Importantly, the association between blood NfL levels and cognitive impairment at follow-up was lost after adjustment for cognitive performance at baseline [28]. In a recent study, Stokowska et al. addressed the association between blood levels of NfL in the late phase after stroke (10 months to 5 years after stroke) and impairment in balance, gait and cognition. Further, the authors explored the utility of blood NfL as a predictor of functional change (worsening or improvement) in late-phase stroke survivors [31]. The study found that similar to the acute phase, even in the late phase after stroke, the blood NfL levels were associated with more pronounced physical and cognitive impairment, and these associations remained significant after adjustment for age [31]. There was no association between blood NfL levels and worsening in any of the parameters assessed at follow-up 3 and 9 months later. Surprisingly, however, that study demonstrated not only that late-phase stroke survivors with higher plasma NfL levels can improve both physically and cognitively, but also that elevated blood NfL levels are a positive predictor of functional improvement in the late phase after stroke [31] (Figure 1A,B). For comparison with the levels of NfL in the acute and post-acute phases described above, the blood NfL levels reported in this study ranged between 5 and 66 pg/mL of plasma, with a median of 18 pg/mL, i.e., were normal to moderately elevated. Given that all associations between blood NfL levels and improvement withstood the correction for the baseline (trial entry) level of impairment, the level of impairment was not the sole driver of the associations. The superiority of blood NfL compared to baseline level of impairment in improvement prediction was further supported by a multivariable regression model in which the difference in the blood NfL levels showed the largest effect on determining the probability of functional improvement in balance and gait [31]. Further, in the late phase after stroke, the blood NfL levels were a more important predictor of improvement than the NIH Stroke Scale score assessed at this time point [31] (Figure 1A,B). It is also noteworthy that the predictive value of blood NfL levels for improvement in balance and gait was independent of 12-week multimodal rehabilitative interventions, namely, rhythm- and music-based therapy or horse-riding therapy, which some of the study participants received. In contrast to the negative findings reported in the abovementioned longitudinal study on the value of blood NfL for the prediction of cognitive impairment in small-vessel disease patients [28], the study by Stokowska et al. found that elevated blood NfL levels were a positive predictor of cognitive improvement, but only in the subgroup of individuals who received rhythm- and music-based therapy, a multimodal neurorehabilitative intervention which predominantly targets the cognitive domain [11] (Figure 1C). Jointly, these findings support the contention that moderately elevated blood NfL levels may capture the improvement potential of stroke survivors and thus aid in predicting the effectiveness of neurorehabilitation. This type of prediction may be particularly useful for the improvement in cognitive functions and for interventions specifically targeting learning and memory.

## 5. The Non-Axonal Functions of NfL and the Aspect of Time after Stroke

**Figure 2:**
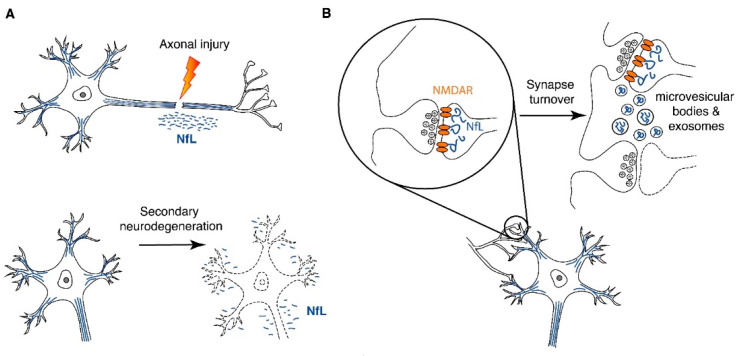
NfL release from neurons after stroke. (**A**) NfL (or NfL-derived peptides) can be released from neurons to the extracellular space as a consequence of direct axonal injury or secondary neurodegeneration. (**B**) NfL plays important role in regulating neurotransmission and other aspects of synaptic function. Microvesicular bodies or endosome-derived exosomes were proposed as hypothetical routes of the active release of NfL or NfL-derived peptides from neurons. NfL release by neurons may reflect synaptic turnover and adaptive plasticity. The exact mechanisms through which NfL or NfL degradation products reach the cerebrospinal fluid and the blood compartment are not known, but the intramural peri-arterial drainage pathway [35] and the glymphatic system [36] that drain peptides and proteins from the extracellular space of the brain to the lymphatic system and blood present conceivable routes.

As described above, the interpretation of elevated blood NfL levels seems to be affected by the time since the incident stroke. The distinction between the predictive value of the blood NfL levels in the acute versus the chronic phase after stroke is also apparent with regard to the associations with stroke recurrence. While in blood samples obtained at 6 months after initial ischemic stroke higher NfL levels were associated with the detection of a new ischemic lesion [24], plasma NfL levels assessed at 10 months to 5 years after stroke did not show association with multiple ischemic stroke events or the time since the last stroke [31]. The intriguing difference between the acute and late phases after stroke in outcome and improvement prediction by higher blood NfL levels raises an important question: can NfL be released into the extracellular space by mechanisms other than neuronal damage? If so, are the mechanisms of NfL release affected by the time after injury? While substantial amount of experimental work is required to fully answer this question, the fact that NfL is abundant also at the synapse offers at least a partial answer. Indeed, NfL from the non-axonal compartment has recently been proposed as a major contributor to the NfL detected in the cerebrospinal fluid and peripheral blood [30]. The mechanisms through which NfL, or peptides derived from NfL through partial degradation in the neuron, are released from neurons are currently only speculative, with microvesicular bodies or smaller endosome-derived exosomes as the hypothetical routes [30] (Figure 2). It is noteworthy that the short NfL-containing neurofilaments in synaptic terminals, in particular, in the postsynaptic compartments, play a role in controlling synaptic function and neurotransmission [30]. For example, synaptic NfL interacts directly with the GluN1 subunit of (N-methyl-D-aspartate) NMDA receptors, increases its surface abundance [32] and blocks its ubiquitination [33]. In this way, NfL may play an important role in stabilizing NMDA receptors in the cell membrane. In further support of the importance of NfL for synaptic function, NfL-deficient mice have reduced dendritic spine density, reduced GluN1 protein levels, elevated ubiquitin-dependent turnover of GluN1 and hippocampal glutamate and reduced hippocampal long-term potentiation [34]. Considering the abundance and function of neurofilament proteins at the synapse, synaptic turnover or synaptic damage in healthy individuals and in the context of neurodegeneration, respectively, may therefore substantially contribute to NfL release into the extraneuronal space, cerebrospinal fluid and systemic circulation.

**Figure 3:**
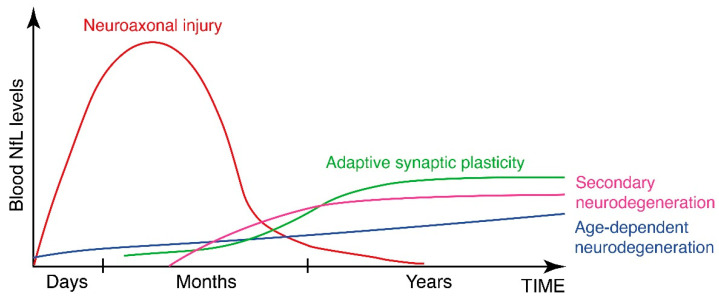
A model of the relative contribution of the different release mechanisms to the blood NfL levels after stroke and how they may change over time. After stroke, blood NfL levels reflect the release of NfL or NfL-derived peptides from injured axons, damaged synapses and age-dependent neurodegenerative processes as well as adaptive plasticity-related synaptic turnover. Whereas in the acute phase after stroke, the axons are the main source of blood NfL, synaptic turnover and secondary neurodegeneration are likely to be major contributors to blood NfL levels in the late phase after stroke.

There is ample experimental and clinical evidence for stroke-induced neural plasticity, both in the peri-infarct region and in the more remote parts of the brain, and its role in functional recovery. This adaptive injury-induced and functional improvement-promoting plasticity is based on the same cellular and molecular mechanisms that are employed for normal learning [3], including synaptic and axonal plasticity. It is possible that blood NfL levels in the post-acute and late phase after stroke represent the summation of two concurrent but very distinct processes of NfL release, namely neuroaxonal injury and synaptic damage, that are associated with secondary neurodegeneration, and adaptive neural plasticity (Figure 3). In the late phase after stroke, the interpretation of elevated blood levels of NfL should not be therefore limited to the extent of injury or neurodegeneration.

## 6. Conclusions

The currently available evidence shows that, while in the acute phase after stroke high blood levels of NfL seem to reliably reflect the extent of neuronal injury, in the late phase after stroke elevated NfL levels in the blood may serve as a biomarker of adaptive neural plasticity and a positive predictor of functional improvement and, therefore, also aid in predicting the effectiveness of neurorehabilitation. This concept deserves to be explored in future clinical studies.

## Data Availability

Not applicable.
